# Between Fajr and Isha: Understanding Sleep Dynamics in Islamic Prayer Timings and Astronomical Considerations

**DOI:** 10.7759/cureus.58911

**Published:** 2024-04-24

**Authors:** Bilal Irfan, Aneela Yaqoob

**Affiliations:** 1 Center for Bioethics, Harvard Medical School, Boston, USA; 2 Michigan Alzheimer's Disease Center, University of Michigan, Ann Arbor, USA; 3 Infectious Diseases, Beaumont Hospital, Taylor, USA

**Keywords:** prayer timing, sleep health, astronomical considerations, salah, islamic prayer

## Abstract

The interplay between Islamic prayer timings, particularly Fajr (the predawn prayer) and Isha (the night prayer), and sleep health is a critical area of study, revealing the significant impact of these religious practices on sleep patterns. Astronomical calculations used to determine these prayer times involve observing the sun's position relative to the horizon at specific degrees of twilight, which can vary, potentially leading to differences in sleep duration and quality among Muslims. This variation is influenced by factors like geography and seasonality, especially in regions far from the equator where daylight hours shift dramatically. For instance, during longer summer days in the northern latitudes, the time for Isha can extend late into the night, potentially shortening sleep if adherents rise early for Fajr. Conversely, in winter, earlier nightfalls may allow for longer sleep periods before Fajr. It is important to highlight the challenges Muslims face in balancing their spiritual obligations with the need for restful sleep. The discussion extends to the implications for healthcare providers, who must navigate cultural sensitivities and promote sleep hygiene within the framework of Islamic practice by suggesting flexible sleep schedules, the strategic use of naps, and education on the importance of maintaining consistent sleep routines.

## Editorial

The intertwining of religious practices with daily routines has profound implications for sleep health, particularly among Muslims, whose prayer timings are closely tied to the astronomical clock. Islamic jurisprudence dictates five daily prayers at specific times, with the Fajr and Isha prayers closely linked to twilight periods, which are astronomically determined by the sun’s position relative to the horizon. However, the exact timing for these prayers is subject to interpretative differences based on various degrees of twilight, leading to variances that can significantly impact sleep duration and quality. Astronomical twilight is categorized into civil, nautical, and astronomical phases, corresponding to different solar depression angles: 6°, 12°, and 18° below the horizon, respectively. These scientific measurements, however, do not always align with the Islamic definitions of dawn and dusk. Islamic scholars often adopt angles between 12° and 18° for Fajr and Isha prayers, but this range can vary based on local observations, geographical location, and other factors such as altitude and atmospheric conditions [1]. Empirical evidence collected from studies in regions such as Scandinavia and North America could serve to underscore how these variances in prayer timings can significantly impact sleep patterns, necessitating tailored health advisories for Muslim populations.

These variances in prayer timings can lead to significant differences in the sleep patterns of Muslims. For instance, the use of an 18° angle for Fajr in North America may result in prayer times that are considerably earlier than those calculated with a 15° angle, potentially shortening the sleep window if individuals awake for Fajr and do not return to sleep. In cities like Anaheim, California, the difference between using 18° and 12° for Fajr can be as much as an hour, which is substantial considering the recommended 7-9 hours of sleep per night for adults. Experts in Islamic jurisprudence and sleep science could suggest that adopting standardized prayer timing guidelines could help mitigate sleep disruptions, while also emphasizing the importance of local astronomical data combined with sound religious interpretations to optimize sleep health among Muslims.

Furthermore, the seasonal variation in day length, especially in regions far from the equator, exacerbates these challenges. In such areas, the time difference for prayer between summer and winter can further disrupt regular sleep patterns, necessitating a flexible approach to managing sleep hygiene among Muslim populations. From a medical perspective, it is crucial to acknowledge the potential impact of these religious practices on sleep health. Sleep is vital for various bodily functions, including cognitive processes, emotional regulation, and overall physical health [2]. Disruptions in sleep patterns, such as those that may occur from early morning or late-night prayer times, can lead to sleep deprivation, mood disturbances, and a decline in daytime functioning when total sleep duration is not maintained [3].

Healthcare providers need to consider these aspects when advising Muslim patients on sleep health. A comprehensive approach that respects religious practices while promoting good sleep hygiene is essential. This may include discussing the possibility of taking short naps to compensate for sleep loss, maintaining consistent sleep routines, and using light exposure to adjust the body's internal clock. Moreover, community-based studies and observations should be encouraged to derive localized recommendations that can help minimize sleep disruption. In addition to individual sleep health, the communal aspect of prayer in Islam, particularly the congregational prayers held at mosques, plays a significant role in shaping sleep patterns. The social and spiritual significance of these gatherings may influence individuals' willingness to adjust their sleep schedules [4]. Thus, community leaders and mosques can play a pivotal role in promoting awareness about the importance of sleep and facilitating schedules that harmonize prayer times with healthy sleep practices.

Technological advancements have led to the development of various apps and tools to calculate prayer times. While these technologies offer convenience, they also pose challenges due to the differing calculation methods they employ, often leading to confusion among users. Educating the Muslim community about these tools, the basis of their calculations, and how to select the most appropriate method for their locale is another crucial step in managing the interplay between prayer timings and sleep health. The relationship between sleep health and prayer timings in Islam is a critical area for both religious and health scholars. The variation in astronomical calculations for prayer times and the direct impact these variations have on sleep duration and quality are significant. Studies have shown that sleep deprivation and irregular sleep patterns can exacerbate health issues such as cardiovascular diseases, cognitive impairments, and metabolic disorders, which are prevalent in many Muslim-majority countries [5].

The complexities of defining twilight in Islamic jurisprudence further complicate the issue. While civil, nautical, and astronomical twilight phases serve as benchmarks in scientific communities, the Islamic definition of dawn and dusk, essential for determining Fajr and Isha prayers, does not always coincide with these scientific phases. This discrepancy necessitates a more flexible and region-specific approach in establishing recommendations for better sleep health among Muslims. In addition to the scientific and religious considerations, cultural factors also play a crucial role in shaping the sleep patterns of Muslim communities. For example, in many Middle Eastern and North African countries, it is common to have late-night activities and social gatherings, further delaying sleep time, especially during Ramadan. The cultural norms and lifestyle choices, combined with the religious obligation of nightly prayers, can lead to significant reductions in sleep duration and alterations in sleep architecture.

Healthcare professionals working with Muslim populations need to be aware of these dynamics to provide culturally sensitive and practical advice. Promoting awareness about the importance of sleep, understanding the implications of prayer timings, and encouraging adherence to sleep hygiene practices can contribute to improved sleep health in these communities. Sleep education programs tailored for Muslim populations can address the unique challenges posed by prayer timings. These programs should include information on the science of sleep, the impact of sleep deprivation, and practical tips for aligning religious practices with healthy sleep habits. Collaboration between healthcare providers, religious authorities, and community leaders is essential to develop and disseminate these educational initiatives effectively.

The sleep health of Muslims is intricately linked to the timing of prayers, governed by Islamic jurisprudence with a view of astronomical calculations. As the global Muslim population navigates the challenges of modern living, including the demands of maintaining prayer times, healthcare providers, community leaders, and technology developers must collaboratively ensure that sleep, a fundamental pillar of health, is not compromised. Recognizing and addressing the diverse factors that influence prayer timings can lead to more informed, health-conscious decisions that respect religious practices while promoting overall well-being. This balanced approach is essential in a world where faith and health are increasingly intersecting domains.
